# Protective Effect of Perilla Seed Meal and Perilla Seed Extract against Dextran Sulfate Sodium-Induced Ulcerative Colitis through Suppressing Inflammatory Cytokines in Mice

**DOI:** 10.3390/molecules29091940

**Published:** 2024-04-24

**Authors:** Natticha Sumneang, Komsak Pintha, Sarawut Kongkarnka, Maitree Suttajit, Napapan Kangwan

**Affiliations:** 1 Department of Medical Science, School of Medicine, Walailak University, Nakhon Si Thammarat 80160, Thailand; natticha.su@wu.ac.th; 2 Research Center in Tropical Pathobiology, Walailak University, Nakhon Si Thammarat 80160, Thailand; 3 Division of Biochemistry, School of Medical Sciences, University of Phayao, Phayao 56000, Thailand; komsakjo@gmail.com (K.P.); maitree.suttajit@gmail.com (M.S.); 4 Department of Pathology, Faculty of Medicine, Chiang Mai University, Chiang Mai 50200, Thailand; srawutzi@gmail.com; 5 Division of Physiology, School of Medical Sciences, University of Phayao, Phayao 56000, Thailand

**Keywords:** rosmarinic acid, ulcerative colitis, perilla seed meal, perilla seed, anti-inflammation

## Abstract

An excessive inflammatory response of the gastrointestinal tract is recognized as one of the major contributors to ulcerative colitis (UC). Despite this, effective preventive approaches for UC remain limited. Rosmarinic acid (RA), an enriched fraction from *Perilla frutescens*, has been shown to exert beneficial effects on disease-related inflammatory disorders. However, RA-enriched perilla seed meal (RAPSM) and perilla seed (RAPS) extracts have not been investigated in dextran sulfate sodium (DSS)-induced UC in mice. RAPSM and RAPS were extracted using the solvent-partitioning method and analyzed with high-pressure liquid chromatography (HPLC). Mice with UC induced using 2.5% DSS for 7 days were pretreated with RAPSM and RAPS (50, 250, 500 mg/kg). Then, the clinical manifestation, colonic histopathology, and serum proinflammatory cytokines were determined. Indeed, DSS-induced UC mice exhibited colonic pathological defects including an impaired colon structure, colon length shortening, and increased serum proinflammatory cytokines. However, RAPSM and RAPS had a protective effect at all doses by attenuating colonic pathology in DSS-induced UC mice, potentially through the suppression of proinflammatory cytokines. Concentrations of 50 mg/kg of RAPSM and RAPS were sufficient to achieve a beneficial effect in UC mice. This suggests that RAPSM and RAPS have a preventive effect against DSS-induced UC, potentially through alleviating inflammatory responses and relieving severe inflammation in the colon.

## 1. Introduction

Concern over the prevalence of ulcerative colitis (UC) has grown significantly on a global scale [1,2]. UC is recognized as a substantial contributor not only to morbidity but also to socioeconomic burden [2]. According to previous reports, its incidence ranges from 9 to 20 per 100,000 person years in the West and 4.6 per 100,000 person years in East Asia [3,4]. Predictions suggest that the large population of the Asia-Pacific region will cause the disease burden of UC to soon surpass that of the West [5,6]. Additionally, it has been reported that patients with UC have an increased risk of developing colorectal cancer [7,8].

UC is one of the main types of inflammatory bowel disease (IBD) that primarily affects the colon and rectum [9]. Although the specific etiology of UC is still unclear, inflammation plays a pivotal role in the onset, development, progression, and recurrence of UC [10]. Several studies have highlighted the significance of proinflammatory cytokines such as tumor necrosis factor-alpha (TNF-α), interleukin-6 (IL-6), and interleukin-1 beta (IL-1β), which are strongly associated with a disruption of the intestinal barrier and intestinal epithelial dysfunction [9,11,12]. Furthermore, the transcription factor nuclear factor-kappa B (NF-κB) has also been implicated in UC development [12]. Previous research has shown that activating NF-kB in UC causes TNF-α, IL-6, and IL-1β expression, which leads to the development of severe clinical manifestations, such as a loss of body weight (BW), abdominal pain, and bloody diarrhea [11,13,14]. Currently, sulfasalazine (SSZ) is commonly used as a first-line therapy in UC patients due to its anti-inflammatory effect and its approximately 50% remission rate [3,15]. However, prolonged treatment with SSZ is associated with side effects attributed to the sulfapyridine molecules [16]. Hence, it is important to conduct research and innovate therapeutic approaches that effectively reduce complications and minimize adverse effects in UC patients.

Recently, there have been developments in the attempt to explore alternative treatment regimens in UC [11,17,18,19]. *Perilla frutescens* L. Britton, commonly known as perilla, is a traditional herb widely found in East Asian countries, including China, Korea, Japan, Vietnam, and Thailand [17,20,21,22,23]. Previous studies have demonstrated that perilla extraction from seeds and leaves can provide various bioactive compounds, such as, α-linolenic acid, apigenin, luteolin, and rosmarinic acid (RA) [18,22,23,24,25,26]. Of these biological compounds, RA, one of the phenolic constituents, has been reported to be abundant in perilla and to exhibit various biological efficacies, including antioxidant, antiallergic, antimicrobial, anticancer, and anti-inflammatory properties [23,26,27,28,29,30,31,32,33,34]. In addition, it has been reported to exert beneficial protective effects in rodent disease models, including allergic asthma and UC [25,34,35,36].

Perilla seeds (PSs) are known for their richness in essential fatty acids, including omega-3 and omega-6, as well as their high content of RA [18,37,38,39]. They are often used as a traditional remedy to treat various illnesses, such as asthma, allergies, abdominal pain, and digestion disorders [40]. They are also popular in Northern Thailand, where they are mixed with sticky rice, which is known as Kao-Nuk-Nga. Furthermore, perilla seed meal (PSM), a by-product of perilla seed oil production commonly used as a source of protein in animal feed, has also been reported to be rich in RA [41,42]. However, the potential benefits of RA-enriched PSM and PS extracts (RAPSM and RAPS, respectively) for UC in mouse models have never been investigated. The aim of this study was to investigate the beneficial effects of RAPSM and RAPS in terms of protection against dextran sulfate sodium (DSS)-induced UC in mice. Therefore, this study hypothesized that RAPSM and RAPS would ameliorate the pathological inflammatory response in DSS-induced UC mice.

## 2. Results

### 2.1. Phytochemical Determination of PSM and PS Extracts

**Figure 1:**
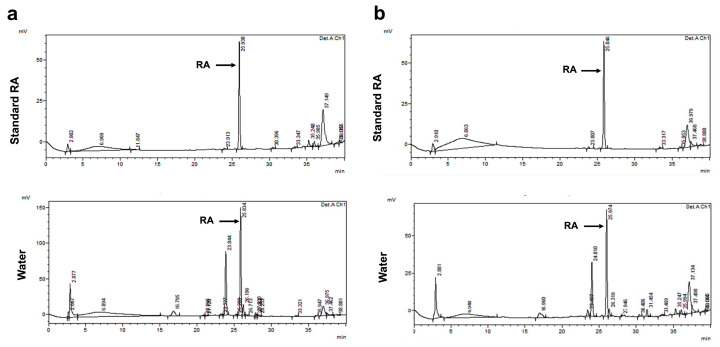
HPLC chromatographic separation of rosmarinic acid-enriched perilla extracts from using partition-extraction in water. (**a**) RAPSM and (**b**) RAPS. HPLC: high-performance liquid chromatography; RAPSM: rosmarinic acid-enriched perilla seed meal extract; and RAPS: rosmarinic acid-enriched perilla seed extract.

The results showed that the highest RA content was obtained from water fractions (47.41 ± 2.08 and 23.02 ± 0.82 mg/g extract) of PSM and PS extracts compared to hexane (Hex) (5.81 ± 0.09 and 1.41 ± 0.0 mg/g extract) and dichloromethane (DCM) (4.73 ± 0.05 and 6.31 ± 0.02 mg/g extract) (Table 1, Figure 1). Therefore, in this study, the water fractions with the highest RA content from the PSM and PS extractions were used for the in vivo study in all experiments. Furthermore, the total phenolic content (TPC) in water fractions of PSM and PS were 51.64 ± 0.10 and 104.71 ± 6.06 mg GAE/g extract, respectively. The total flavonoid content (TFC) in water fractions of PSM and PS were 90.23 ± 0.59 and 35.60 ± 9.24 mg CE/g extract, respectively.

### 2.2. Effect of RAPSM and RAPS on Symptomatic Manifestations in DSS-Induced UC Mice

**Figure 2:**
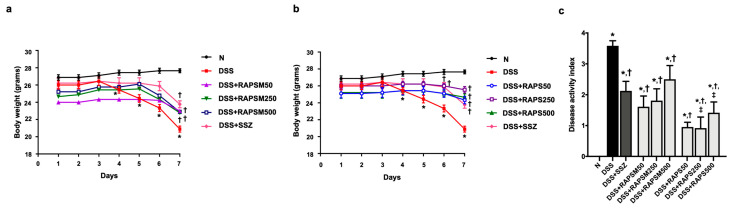
RAPSM and RAPS reduced symptomatic manifestations in DSS-induced UC mice. (**a**) Effect of RAPSM on BW, (**b**) effect of RAPS on BW, and (**c**) DAI score. Data are expressed as ± S.E.M (*n* = 8 per group). * *p* < 0.05 vs. N; † *p* < 0.05 vs. DSS; and ‡ *p* < 0.05 vs. DSS + RAPSM at the same dose. RAPSM: rosmarinic acid-enriched perilla seed meal extract; RAPS: rosmarinic acid-enriched perilla seed extract; DSS: dextran sulfate sodium; UC: ulcerative colitis; BW: body weight; and DAI: disease activity index.

Mice pretreated with the vehicle demonstrated a loss of body weight (BW) after 4 days of DSS administration (DSS group) (Figure 2a,b). Additionally, the DSS-treated mice exhibited symptoms of diarrhea and bloody stools, resulting in an increased DAI score compared to the mice pretreated with the vehicle alone (N group) (Figure 2c). These findings indicated that treatment with DSS successfully induced acute UC in mice.

Pretreatment with all doses of RAPSM gradually attenuated the reduction in BW observed in DSS-treated mice (Figure 2a). Similarly, administration of all doses of RAPS resulted in a gradual increase in BW compared to the DSS group (Figure 2b). This suggests that RAPSM and RAPS improved the body weight of DSS-induced UC mice. In addition, the mice pretreated with both RAPSM and RAPS at 50, 250, and 500 mg/kg showed a significant decrease in DAI score compared to the DSS group (Figure 2c). Although administration of RAPS at doses of 250 and 500 mg/kg showed greater efficacy in reducing the DAI score than administration of RAPSM at the same dose, RAPSM and RAPS at a dose of 50 mg/kg showed a comparable ability to reduce the DAI score to a similar extent in DSS-induced UC mice (Figure 2c). This suggests that RAPSM and RAPS reduced the severity of colitis in DSS-induced UC mice. These findings suggested that pretreatment with RAPSM and RAPS was effective in preventing and attenuating symptomatic manifestations in DSS-induced UC mice. In addition, SSZ also increased BW and reduced the DAI score in DSS-induced UC mice (Figure 2a–c).

### 2.3. Effect of RAPSM and RAPS on the Shortening of Colon Length in DSS-Induced UC Mice

**Figure 3:**
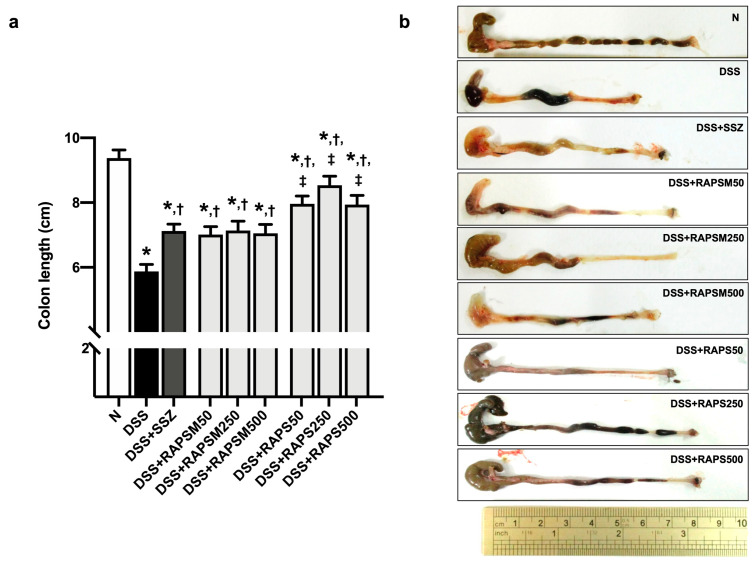
RAPSM and RAPS decreased the shortening of colon length in DSS-induced UC mice. (**a**) Colon length and (**b**) representative picture of colon gross appearances. Data are expressed as ± S.E.M (*n* = 8 per group). * *p* < 0.05 vs. N; † *p* < 0.05 vs. DSS; and ‡ *p* < 0.05 vs. DSS + RAPSM at the same dose. RAPSM: rosmarinic acid-enriched perilla seed meal extract; RAPS: rosmarinic acid-enriched perilla seed extract; DSS: dextran sulfate sodium; and UC: ulcerative colitis.

Mice treated with DSS had a shortening of the colon, suggesting that UC conditions were observed in this group (Figure 3). In comparison, pretreatment with RAPSM and RAPS at all doses significantly reduced colon length shortening (Figure 3a). At similar doses, pretreatment with RAPS showed better efficacy than RAPSM in alleviating colon length shortening at all doses (Figure 3a). However, pretreatment with RAPSM and RAPS at 50 mg/kg resulted in equally improved colon lengths compared to the DSS group (Figure 3a).

Similarly, treatment with sulfasalazine (SSZ) reduced colon length shortening in DSS-induced UC mice, consistent with the administration of all doses of RAPSM and RAPS. The representative gross appearance of colon length is shown in Figure 3b. The results suggested that pretreatment with RAPSM and RAPS prevented the severity of UC by reducing colon length shortening.

### 2.4. Effect of RAPSM and RAPS on Histopathological Damage in DSS-Induced UC Mice

**Figure 4:**
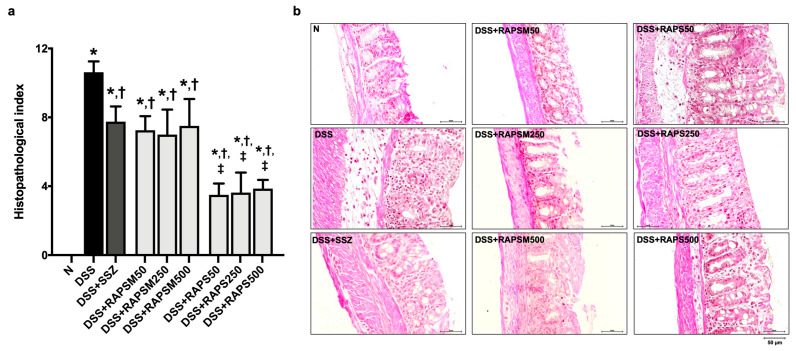
RAPSM and RAPS alleviated histopathological damage in DSS-induced UC mice. (**a**) Histopathological index and (**b**) Representative picture of colon tissue stained with H&E (scale bar = 50 µm). Data are expressed as ±S.E.M (*n* = 8 per group). * *p* < 0.05 vs. N; † *p* < 0.05 vs. DSS; and ‡ *p* < 0.05 vs. DSS + RAPSM at the same dose. RAPSM: rosmarinic acid-enriched perilla seed meal extract; RAPS: rosmarinic acid-enriched perilla seed extract; DSS: dextran sulfate sodium; UC: ulcerative colitis; and H&E: hematoxylin and eosin.

The colon tissue presented architectural abnormalities in DSS-treated mice, including crypt distortion, surface epithelial cell damage, and edematous submucosa (Figure 4a,b). This observation corresponded to a significant increase in the colon histopathological index in the DSS group compared to the N group (Figure 4a).

Pretreatment with RAPSM and RAPS at all doses after 21 days alleviated the histopathological changes in the colon of the DSS-treated mice, as evidenced by an increase in the histopathological index compared to the DSS group (Figure 4a,b). In addition, pretreatment with RAPS at doses of 50, 250, and 500 mg/kg resulted in an enhanced reduction in the histological index than pretreatment with similar doses of RAPSM (Figure 4a). Although RAPS had greater efficacy than RAPSM, both RAPSM and RAPS at a dose of 50 mg/kg were sufficient to reduce the histopathological index to a similar extent as pretreatment with all doses in the DSS-treated mice (Figure 4a). This suggests that RAPSM and RAPS have a protective effect in ameliorating histological changes in DSS-induced UC mice. The representative pictures of the colon structure are shown in Figure 4b. SSZ also alleviated the colon histological changes in DSS-treated mice, similarly to the pretreatments with RAPSM and RAPS at all doses (Figure 4a,b).

### 2.5. Effect of RAPSM and RAPS on Proinflammatory Cytokines in DSS-Induced UC Mice

**Figure 5:**
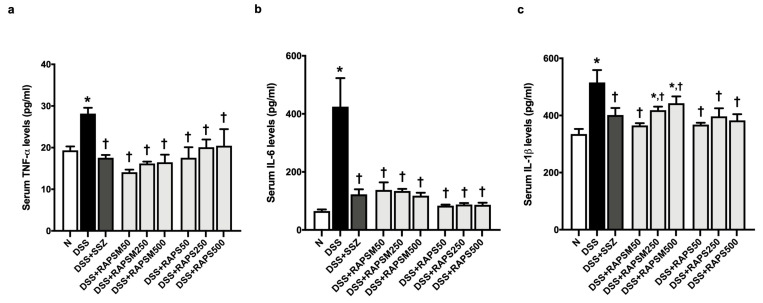
RAPSM and RAPS decreased proinflammatory cytokines in DSS-induced UC mice. (**a**) Serum TNF-α levels, (**b**) serum IL-6 levels, and (**c**) serum IL-1β levels. Data are expressed as ±S.E.M (*n* = 8 per group). * *p* < 0.05 vs. N, and † *p* < 0.05 vs. DSS. RAPSM: rosmarinic acid-enriched perilla seed meal extract; RAPS: rosmarinic acid-enriched perilla seed extract; DSS: dextran sulfate sodium; UC: ulcerative colitis; TNF-α: tumor necrosis factor-alpha; IL-6: interleukin-6; and IL-1β: interleukin-1 beta.

The results showed that serum TNF-α, IL-6, and IL-1β levels were significantly elevated in the DSS-treated mice compared to the mice in the N group (Figure 5a–c). This indicates that UC was successfully induced in mice through an increase in proinflammatory cytokines after 7 days of treatment with DSS (Figure 5a–c). Pretreatment with RAPSM and RAPS at all doses reduced TNF-α and IL-6 in the serum of DSS-treated mice to normal levels (Figure 5a,b). Pretreatment with RAPS reduced the serum IL-1β levels at all doses; however, only pretreatment with RAPSM at a dose of 50 mg/kg reduced serum IL-1β to a normal level in the DSS-treated mice (Figure 5c). Pretreatment with RAPSM at doses of 250 and 500 mg/kg decreased serum IL-1β levels in these mice (Figure 5c). The data suggest that pretreatment with RAPSM and RAPS at a dose of 50 mg/kg was sufficient to restore the serum TNF-α, IL-6, and IL-1β levels of the DSS-treated mice to normal levels (Figure 5a–c). Treatment with SSZ decreased the levels of serum TNF-α, IL-6, and IL-1β in the DSS-treated mice (Figure 5a–c).

## 3. Discussion

The major findings from this study are as follows: (1) PSM and PS extracts rich in RA exert a protective effect on DSS-induced UC by suppressing proinflammatory cytokines and alleviating colon pathology in mice. (2) RAPSM and RAPS, at least at 50 mg/kg, were sufficient to achieve a beneficial effect in preventing and attenuating proinflammatory cytokines and symptomatic manifestations at the same level in DSS-induced UC mice; interestingly, RAPS initially showed greater efficacy at a higher level. The data are summarized in Table 2.

DSS-induced UC is an acceptable experimental inflammatory model for evaluating the function of the molecular pathology of the colon, and it reflects the clinical symptoms and histological changes observed in humans, including decreases in BW and colon length, and an increase in the DAI score [43,44,45]. In principle, DSS causes lethal colitis, as its negatively charged sulfated polysaccharides directly contribute to the erosion of the intestinal epithelial cells [19,44,45]. This is followed by an increased permeability of the colonic epithelium, enabling laminar microbes to invade the laminar propria [44,46]. In addition, previous studies have demonstrated that a disruption of the intestinal epithelium recruits immune cells, such as neutrophils and macrophages, to secrete proinflammatory cytokines via activation of the NF-kB signaling pathway [19,43,44,45]. As a result, the increased production of proinflammatory cytokines damages intestinal epithelial cells, which eventually leads to colitis [19,44,46]. These findings are consistent with those of this study, where the administration of 2.5% DSS successfully induced UC in mice as evidenced by decreased BW, shortened colon length, increased DAI score, and an architectural distortion of the colon. In addition, the role of proinflammatory cytokines has been demonstrated under UC conditions [19,43], which correlates with the results of this study showing that the serum levels of TNF-α, IL-6, and IL-1β were elevated in the DSS-treated mice. Currently, SSZ is often considered as a first-line agent in the treatment of colitis due to its ability to combat inflammation by suppressing the transcription of NF-kB-responsive proinflammatory cytokines [47,48]. In this study, it was shown that treatment with SSZ attenuated these clinical symptoms and alleviated DSS-induced colitis in mice.

Recently, several studies have reported that extracts of *Perilla frutescens* from various plant parts, especially leaves, stems, and seeds, can be used to treat UC conditions in rodent models [18,25,49]. It contains various phytochemicals with bioactive compounds, including phenolic acids, flavonoids, and anthocyanins [23,50,51,52,53,54,55]. Interestingly, it was demonstrated that among the bioactive compounds from perilla leaves, stems, and seeds, RA has the dominant phenolic activity with a strong anti-inflammatory response [25,50,51,52,53]. The RA extracted from perilla leaves has been reported to directly promote naive T cells while suppressing Th17 differentiation, thereby reducing proinflammatory cytokines in colitis mice [25]. At the molecular level, RA could suppress proinflammatory cytokines and inflammatory mediators by inhibiting the NF-kB/STAT3 signaling pathway, which has been confirmed by the use of commercial RA in colitis mice [34]. In addition, Toll-like receptor 4 (TLR4), one of the upstream signaling pathways of NF-kB, was blocked by RA administration to reduce inflammation via a competitive interaction with TLR4 at the binding sites of Arg-264 residue in a disease-related colitis mouse model [56]. In summary, the evidence supports the recommendation of RA as a potent alternative treatment for UC.

Following the promising evidence of the beneficial effects of RA-enriched extraction from various parts of perilla under pathological conditions in in vitro and in vivo models [25,34,40,57,58], this study is the first to show the favorable effect of RA-enriched fractions from PSM, a by-product of perilla seed oil, and RA-enriched fractions from PS in a DSS-induced UC mouse model. A previous study reported by Khanaree et al. demonstrated that RA is a major bioactive compound in the crude extract of PSM and PS [58]. Therefore, this study focused on performing RA-enriched PSM and PS using solvent partitioned via liquid–liquid extraction to improve the purity of RA. Interestingly, it has also shown that treatment with RAPSM at doses of 50, 250, and 500 mg/kg reduced the levels of proinflammatory cytokines, including TNF-α and IL-6, similarly to treatment with RAPS at the same doses in DSS-induced UC mice. Although 50 mg/kg concentrations of RAPSM and RAPS were similarly effective in restoring IL-1β levels to normal in DSS-induced UC mice, RAPSM at doses of 250 and 500 mg/kg reduced IL-1β to slightly less than treatment with RAPS at the same dosages. In this regard, the results showed that the water fraction of PSM contained more RA-enriched extracts than that of PS, while the water fraction of PS had a higher total phenolic content than that of PSM. This implied that apart from RA, another phenolic component of PS played a role in modulating inflammation. As expected, previous studies have reported that 30-dehydroxyl-RA-3-O-glucoside and RA-3-O-glucoside were the dominant phenolics in the extracted PS [24,31,50,59]. Other than RA, PSM and PS extracts also include additional components such as apigenin and luteolin [58]. These substances have been shown to have anticolitic and anti-inflammatory actions [60,61], implying that they may work synergistically with RA to attenuate DSS-induced colitis in animal models. Although Khanaree et al. demonstrated that the levels of apigenin and luteolin were similar in crude extracts of PSM and PS [58], the levels of these components may have differed in this study as they were found to be higher in PS than in PSM. Several factors contribute to differences in the phytochemically and biologically active components of PSM and PS extracts, such as moisture content, duration of maturation, harvest times, environmental conditions, growing climate, seed maturity stage, season, and location [52,58,62,63,64]. However, the benefits are attributed to the significant anti-inflammatory effect of RAPS, which showed a slightly greater reduction in IL-1β levels than RAPSM in this study. Nevertheless, treatments with both RAPSM and RAPS at doses of 50 mg/kg are sufficient to restore proinflammatory cytokines to normal levels in DSS-induced UC mice. RAPSM had a higher content of RA than RAPS and both components demonstrated a beneficial effect at an initial dose of 50 mg/kg; it can be stated that RA has the potential to suppress the inflammatory response in UC.

An excessive inflammatory response has been regarded as a major contributor to the disruption of the properties of colonic mucus in UC conditions [65]. Several studies have demonstrated that RA can suppress inflammatory responses associated with colonic pathology, including mucosal disruption and a loss of intestinal crypts in colitis rodent models [34,57]. Furthermore, they found that RA also relieved weight loss, DAI score, and colon length shortening in colitis conditions [34,57]. Since treatment with RAPSM and RAPS was able to suppress inflammation, in this study it was observed that treatment with RASM and RAPS at doses of 50, 250, and 500 mg/kg reduced colonic pathology in DSS-induced UC mice. In addition, all doses of both treatments with RAPSM and RAPS reduced colon pathology to the same extent in DSS-induced UC mice. Although they contributed equally to the relief of colitis, the effect of RAPS was slightly better than that of RAPSM. These promising results emphasize that extracted PS could be enriched with additional bioactive ingredients that allow its efficacy to surpass that of PSM, as mentioned above. However, the results suggested that RAPSM and RAMS at a dosage of 50 mg/kg could partially improve colon pathology in DSS-induced UC in mice. The findings also demonstrated that treatment with SSZ reduced colonic pathology by improving the DAI score, histologic index, and colon length, and restored proinflammatory cytokines in mice with DSS-induced colitis; however, SSZ did not improve BW in these mice unlike treatment with RAPSM and RAPS. Remarkably, RAPSM and RAPS showed protective effects against DSS-induced colitis by suppressing proinflammatory cytokines and reducing the severity of symptoms in mice. Therefore, RAPSM and RAPS have potential as dietary supplements. This warrants further investigation for the prevention of intestinal inflammatory-related diseases. In addition, although the data demonstrated that RAPSM and RAPS effectively reduced the severity of colitis, further study is required to investigate the mechanisms of RAPSM and RAPS at the molecular level in DSS-induced UC models.

## 4. Materials and Methods

### 4.1. Preparation of PSM and PS Extracts

The PS was collected from Phayao Province, Thailand. The PSM was obtained after pressing for oil extraction. The PSM and PS extracts were prepared by the following previous method [23]. Briefly, the PSM and PS were finely ground into powder, and then extracted with 70% ethanol. After evaporation and lyophilization, the dried ethanolic extracts were then fractionated to Hex/water (1:1), DCM, and water fractions [40]. The obtained dried fractions were stored at −20 °C for further analysis.

### 4.2. Identification of RA and Phytochemical Analysis

The RA content in all fractions was determined using HPLC (Agilent Technologies, Inc., Santa Clara, CA, USA) [23]. The HPLC system was equipped with a C18-EPS Rocket column (53 mm × 7 mm, GRACE). The mobile phase contained a mixture of 0.1% trifluoroacetic acid and acetonitrile with the flow rate set at 1.0 mL/min. The RA was detected at a wavelength of 280 nm.

The determination of TPC was performed using the modified Folin–Ciocalteu colorimetric method [30]. In brief, the sample or standard (as gallic acid) was mixed with Folin–Ciocalteu reagent and 7.5% sodium carbonate and kept in the dark for 15 min. The absorbance at 765 nm was then measured. The TPC was expressed as gallic acid equivalents in milligrams per gram of extract (mg GAE/g extract). In addition, TFC was determined through the colorimetric aluminum chloride method [31]. The sample or standard was added to 5% sodium nitrite for 5 min and then mixed with 10% aluminum chloride and 1 M sodium hydroxide and kept in the dark for 10 min. The absorbance at 510 nm was used to determine the mixture using catechin as a flavonoid standard. The TFC was expressed as milligrams of catechin equivalent per gram of extract (mg CE/g extract).

### 4.3. Animal Experiment and Ethical Approval

**Figure 6:**
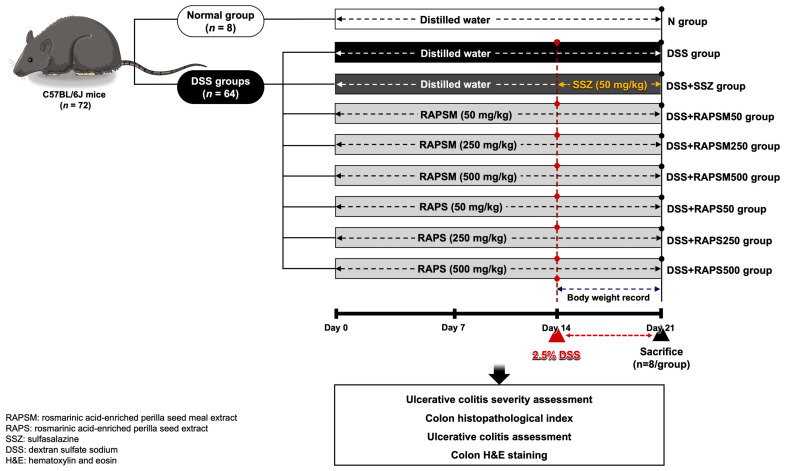
Schematic overview of experimental protocol. RAPSM: rosmarinic acid-enriched perilla seed meal extract; RAPS: rosmarinic acid-enriched perilla seed extract; SSZ: sulfasalazine; and DSS: dextran sulfate sodium.

All the animal experiments were approved by the Institutional Animal Ethics Committee, University of Phayao, Thailand (approval no. 5801040009). Seventy-two male C57BL/6J mice, weighing between 20 and 22 g (six weeks old), were obtained from the National Laboratory Animal Center, Mahidol University (Bangkok, Thailand). All mice were housed in polyethylene cages under specific-pathogen-free conditions. They were kept in a temperature-controlled room set at 25 ± 2 °C with a 12 h dark/light cycle and maintained at a humidity of 50 ± 10% throughout the entire duration of the experimental protocols. After a one-week acclimatization period, the mice were divided into three pretreatments as follows: (1) vehicle (distilled water, *n* = 16), (2) RAPSM (at 50, 250, and 500 mg/kg, *n* = 8 for each dose), and (3) RAPS (at 50, 250, and 500 mg/kg, *n* = 8 for each dose). All treatments were administrated to the mice daily via oral gavage for a duration of 21 days. During the pretreatment, from day 15 to 21, half of the mice in the vehicle (*n* = 8), RAPSM, and RAPS groups were subjected to treatment with 2.5% DSS (molecular weight 36,000–50,000 Da, MP Biomedicals, Solon, OH, USA) in their drinking water for 7 days to induce acute colitis [66]. In this study, SSZ was used as a positive control. An amount of 50 mg/kg of SSZ was co-administered with DSS daily via oral gavage for 7 days. The BW of the mice was monitored daily. At the end of the experimental protocols, the DAI was assessed, and all mice were subsequently euthanized using CO_2_ asphyxiation. Blood samples and colon tissues were collected to determine factors pertaining to inflammation. Additionally, colon length and colon histology were also determined. Figure 6 shows the protocol of this study.

### 4.4. Ulcerative Colitis Severity Assessment

DAI scoring was used to determine UC severity. DAI is a standardized protocol to assess the clinical severity of UC by combining scores for summation of % weight loss, fecal bleeding, and stool consistency divided by 3 [19,67]. The DAI scoring is shown in Table 3.

### 4.5. Histopathological Analysis

Distal colon tissue with a length of 1 cm was preserved in a 10% formalin solution for 24 h before being embedded in paraffin. Then, it was stained with hematoxylin and eosin (H&E) and analyzed using the histopathological index [19,68]. In brief, the colon tissues were graded for the evaluation of the pathohistological index as shown in Table 4 [19,69].

### 4.6. Determination of Proinflammatory Cytokines

The serum levels of proinflammatory cytokines, including TNF-α, IL-1β, and IL-6, were measured using an ELISA kit (BioLegend, San Diego, CA, USA) according to the manufacturer’s instruction. Briefly, 100 µL of capture antibody was added to all wells of a 96-well plate. The plate was sealed and incubated overnight at 4 °C, and then incubated for 30 min at room temperature and washed four times with a washing buffer. Then, Assay Diluent was added to all wells and 50 µL of the diluted standard or the sample was added to each well. The determination was performed in duplicate. The plate was washed after incubation at room temperature with gentle shaking for 2 h. Subsequently, 100 µL of detection antibody was added and the plate was incubated again with gentle shaking at room temperature for 1 h. After that, the plate was washed, and 100 µL of Avidin-HRP was added and incubated for 30 min. Next, the plate was washed and tetramethylbenzidine substrate was added to each well and kept in the dark for 15 min. The color change was determined using a microplate reader (Metertech, Taipei, Taiwan) at 450 nm [66].

### 4.7. Statistical Analysis

Data are expressed as the mean ± standard error of mean (S.E.M). The differences between groups were analyzed using one-way ANOVA followed by the LSD post hoc test. A *p*-value less than 0.05 was considered statistically significant.

## 5. Conclusions

This study is the first to show that treatment with RAPSM and RAPS can significantly alleviate colonic pathology by suppressing the inflammatory response in DSS-induced UC in mice. RAPSM and RAPS at a dosage of 50 mg/kg deserve further consideration as potential therapeutic agents for the treatment of colitis. In addition, the potential of perilla waste products could be increased by using them as a base for the exploration of RA, which could have significant positive implications for the food industry and the medical field.

## Figures and Tables

**Table 1:** RAPSM and RAPS in each fraction.

Fractions	RAPSM	RAPS
Hex	5.81 ± 0.09	1.41 ± 0.01
DCM	4.73 ± 0.05	6.31 ± 0.02
Water	47.41 ± 2.08	23.02 ± 0.82

Data presented as mean ± S.D. RAPSM: rosmarinic acid-enriched perilla seed meal extract (mg/g extract), RAPS: rosmarinic acid-enriched perilla seed extract (mg/g extract), Hex: hexane, and DCM: dichloromethane.

**Table 2:** A summary of the data in this study.

Parameters			Major Findings (vs. N Group)						
	N	DSS	DSS + SSZ	DSS + RAPSM			DSS + RAPS		
				50 mg/kg	250 mg/kg	500 mg/kg	50 mg/kg	250 mg/kg	500 mg/kg
Body weight		↓	↓	↔	↔	↔	↔	↔	↔
DAI score		↑↑	↑	↑	↑	↑	↑ *	↑ *	↑ *
Colon length		↓↓	↓	↓	↓	↓	↓ *	↓ *	↓ *
Histopathological index		↑↑	↑	↑	↑	↑	↑ *	↑ *	↑ *
TNF-α levels		↑	↔	↔	↔	↔	↔	↔	↔
IL-6 levels		↑	↔	↔	↔	↔	↔	↔	↔
IL-1β levels		↑↑	↔	↔	↑	↑	↔	↔	↔

N: normal group; DSS: dextran sodium sulfate-induced ulcerative colitis group; SSZ: sulfasalazine; RAPSM: rosmarinic acid-enriched perilla seed meal extract; RAPS: rosmarinic acid-enriched perilla seed extract; DAI: disease activity index; TNF-α: tumor necrosis factor-alpha; IL-6: interleukin-6; IL-1β: interleukin-1 beta; ↑: an increase; ↓: a decrease; and ↔: no change. * Superior effect compared to RAPSM administration at the same dose.

**Table 3:** Assessment of disease activity index * [19,67].

Score	Weight Loss (%)	Stool Consistency	Gross Bleeding
0	None	Normal stool, well form pellets	No rectal bleeding
1	1.0–5.0	-	-
2	5.1–10.0	Loose stools, pasty stools that do not stick to the anus	Hemoccult positive
3	10.1–15.0	-	-
4	>15.0	Diarrhea, liquid stools that stick to the anus	Visible gross bleeding

* The disease activity index (DAI) = (combined score of weight loss, stool consistency, and gross bleeding)/3.

**Table 4:** Assessment of histopathological index [19,69].

Lesion Criteria	Score	Descriptive Remarks
1. Severity of ulceration/erosion	0	Epithelium intact
	1	Involvement of laminar propria
	2	Involvement of the mucosa
	3	Into colon wall
2. Area affected by intestinal inflammation	0	None
	1	<10%
	2	10%
	3	10–50%
	4	>50%
3. Extension of follicle aggregation	0	None
	1	Mild
	2	Moderate
	3	Severe
4. Edema	0	None
	1	Mild
	2	Moderate
	3	Severe
5. Loss of crypt	0	None
	1	<10%
	2	10%
	3	10–50%
	4	>50%
6. Infiltration of inflammatory cells	0	None
	1	Mild
	2	Moderate
	3	Severe

## Data Availability

Data are contained within the article.

## Abbreviations

UC	Ulcerative colitis
IBD	Inflammatory bowel disease
RA	Rosmarinic acid
RAPSM	Rosmarinic acid-enriched perilla seed meal extract
RAPS	Rosmarinic acid-enriched perilla seed extract
DSS	Dextran sulfate sodium
HPLC	High-pressure liquid chromatography
TNF-α	Tumor necrosis factor-alpha
IL-6	Interleukin-6
IL-1β	Interleukin-1 beta
NF-κB	Nuclear factor-kappa B
BW	Body weight
SSZ	sulfasalazine
PSM	Perilla seed meal
PS	Perilla seed
Hex	Hexane
DCM	Dichloromethane
TPC	Total phenolic content
TFC	Total flavonoid content
DAI	Disease activity index
H&E	Hematoxylin and eosin
TLR4	Toll-like receptor 4
STAT3	Signal transducer and activator of transcription 3
